# Monitoring and detection of leprosy patients in Southwest China: A retrospective study, 2010–2014

**DOI:** 10.1038/s41598-018-29753-4

**Published:** 2018-07-30

**Authors:** Wang Le, Jiang Haiqin, Hao Danfeng, Shi Ying, Zhang Wenyue, Yang Jun, Xiong Li, Shui Tiejun, Shen Limei, Liu Jie, Wang De, Ning Yong, Liu Yangying, Wang Hao, Kuang Yanfei, Li Bin, Yumi Maeda, Malcolm Duthie, Yu Meiwen, Wang Hongsheng, Yan Liangbin, Zhang Guocheng, Wang Baoxi, Gu Heng

**Affiliations:** 1 Institute of Dermatology, Chinese Academy of Medical Sciences & Peking Union Medical College, Nanjing, China; 2 National Center for STD and Leprosy Control, China CDC, Nanjing, China; 3 Jiangsu Key Laboratory of Molecular Biology for Skin Diseases and STIs, Nanjing, China; 4 Yunnan Provincial CDC, Kunming, China; 5 Guizhou Provincial CDC, Guiyang, China; 6 Sichuan Provincial People’s Hospital, Chengdu, China; 7 Hunan Provincial CDC, Changsha, China; 8 Department of Mycobacteriology, Leprosy Research Center, National Institute of Infectious Diseases, Tokyo, Japan; 9 Infectious Disease Research Institute, Seattle, USA

## Abstract

More than 100 counties, mainly in southwest China, report incidence rates of leprosy >1/100,000. The current study analysed the epidemiology of leprosy in southwest China to improve our understanding of the transmission pattern and improve control programs. 207 counties were selected in southwest China. Leprosy patients and their household contacts were recruited. The data from the medical interview and the serological antileprosy antibody of the leprosy patients were analysed. A total of 2,353 new cases of leprosy were interviewed. The distribution of leprosy patients was partly associated with local natural and economic conditions, especially several pocket areas. A total of 53 from 6643 household contacts developed leprosy, and the incidence rate of leprosy in the household contacts was 364/100,000 person-years. We found that NDO-BSA attained higher positive rates than MMP-II and LID-1 regardless of clinical types, disability and infection time in leprosy patients. By means of combination of antigens, 88.4% patients of multibacillary leprosy were detected, in contrast to 59.9% in paucibacillary leprosy. Household contacts should be given close attention for the early diagnosis, disruption of disease transmission and precise control. Applications of serology for multi-antigens were recommended for effective coverage and monitoring in leprosy control.

## Introduction

Leprosy is a chronic infectious disease caused by *Mycobacterium leprae* that can progress to peripheral nerve injury and systematic deformity in untreated individuals^1^. Leprosy remains a significant health problem in several parts of the world and according to the official WHO records, 211,973 new cases were reported globally in the year 2015; China contributed 678 (0.32%) cases (WHO, http://www.who.int/lep/epidemiology/en/). Leprosy was eliminated as a national health concern in China through the successful completion of two distinct control stages. The first stage was conducted between 1950 and 1980 and aimed at controlling the infectious source of the disease, while the second stage, from 1981 to the present day, has focused on bringing prevalence rates <1 case per 100,000 at the county level^2^. Leprosy incidence in China has accordingly decreased in recent years^3^. However, the disease is still a public health problem in several areas in southwest China and it is therefore of interest to define the current characteristics of leprosy as the epidemiology of leprosy has evolved.

Since the recognition by Hunter and Brennan that phenolic glycolipid-I (PGL-I) is a major antigen unique to *M*. *leprae*, PGL-I has been widely used as a target for the serodiagnosis of leprosy^4^. Several additional antigens have now also been identified, such as natural disaccharide-octyl-bovine serum albumin (NDO-BSA), a synthetic second-generation mimetope of native PGL-I, and the protein targets MMP-II and LID-1^5^. By the standard reports available, 241 counties have so far failed to reach the target of a prevalence rate <1 case per 100,000 population at their level, and some 21 counties still have prevalence rates of more than 10 cases per 100,000. Yunnan, Guizhou, Sichuan and Hunan provinces are the main provinces affected and contain 75.5% and 90.5% of the counties with prevalence rates greater than 1 and 10 cases per 100,000, respectively. The 2510 leprosy cases recorded in these four provinces accounted for 52.6% of the total leprosy cases (4775) registered in the whole of China between 2011 and 2015, indicating ongoing *M*. *leprae* transmission in these regions. We have previously evaluated serum antibodies against PGL-I and MMP-II among Chinese leprosy patients and household contacts (HHC), finding them to be more prevalent in multibacillary (MB) than paucibacillary (PB) patients and indicating utility for screening to detect early *M*. *leprae* infection in HHC^4^. It is well known that close contacts of leprosy patients have an increased risk of *M*. *leprae* infection and although it was reported approximately 30% of newly diagnosed leprosy patients in southwest China had a history of contact with known leprosy patients, the incidence rate among household contacts of developing leprosy has not been accurately defined.

To improve our understanding of the current situation, in this report we detailed the demographics and serum antigen-specific antibody responses of leprosy patients in southwest China, and assessed the relationship between clinical presentations, occurrence of disability and antigen-specific serum antibody responses. We also evaluated the incidence rate among HHCs and compared the data generated against the general population.

## Methods

### Study area

**Figure 1:**
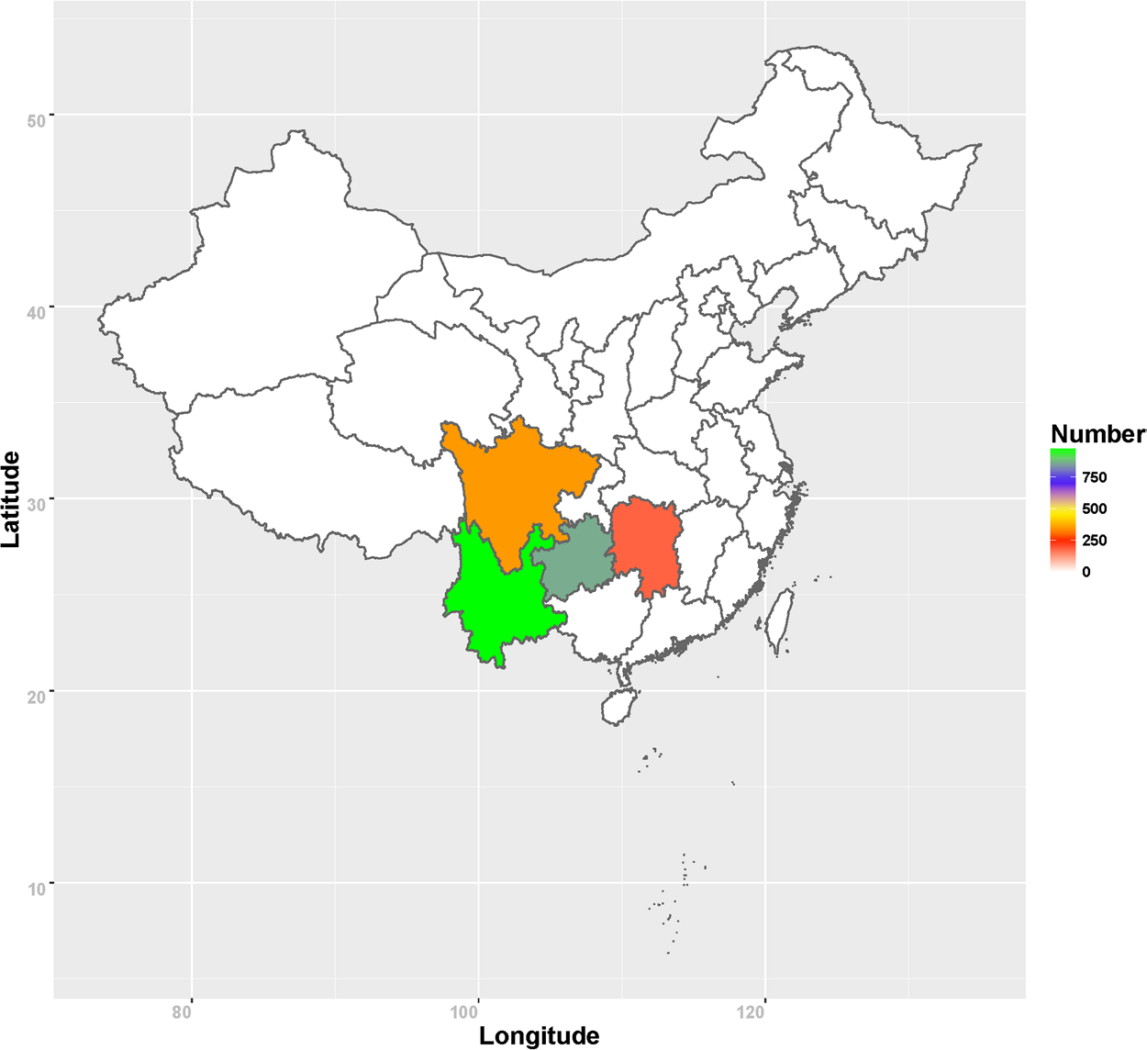
Study areas and geographic distribution of new leprosy cases in southwest China between January 2010 and June 2014. Study areas include four provinces, Yunnan, Guizhou, Sichuan and Hunan, corresponding to pale green, dark green, orange and red colour in southwest China. Green = the most patients, red = the least patients. The map in this figure was generated by means of software R, including maptools, maps and mapdata (Version 3.3.3, https://cran.r-project.org/src/base/R-3/).

A prospective survey was conducted from 2010–2014 in 4 provinces (Yunnan, Guizhou, Sichuan and Hunan), encompassing 207 counties or districts (78 from Yunnan, 55 from Guizhou, 34 from Sichuan and 40 from Hunan). These provinces are mainly located between 20°N and 30°N latitudes and typically have a tropical or subtropical climate (Fig. 1). Most of these districts reported prevalence rates for leprosy of >1/100,000^2^.

### Participants

All leprosy patients who attended clinics to receive diagnosis and treatment during the period January 2010 to June 2014 were included in the study. Family members, or any person living with a patient (healthy household contact; HHC), were also registered and monitored. Persons that had resided with leprosy patients for at least 6 months from 6 years before the initiation of Multidrug therapy (MDT), or up to 1 month after its completion, were also usually considered as HHC.

### Clinical data and blood sample collection

Data were obtained from local staff that were actively monitoring leprosy patients and thereby involved in leprosy eradication programs in the concerned provinces. The personal data collected included name, age, distribution and date of diagnosis and clinical information such as clinical presentation, grade of deformities and disability and are summarized in Table 1. Patients were diagnosed as having leprosy by initial clinical evaluation followed by further classification based on clinical manifestations, slit skin smears and histopathological examinations. HHC were examined and monitored for the cardinal signs and symptoms of leprosy, with potential disease progression also addressed. Blood was collected from all the leprosy patients after obtaining an informed written consent. Sera were prepared and stored at −20 °C until use.

**Table 1:** Clinical characteristics between different gender groups.

	Total		Male(N = 1664)		Female(N = 689)		p-value
	N	%	N	%	N	%	
Age distribution							0.137
<15	82	3.5	53	3.2	29	4.2	
15–29	506	21.5	349	21.0	157	22.8	
30–44	769	32.7	566	34.0	203	29.5	
45–59	624	26.5	428	25.7	196	28.4	
≥60	372	15.8	268	16.1	104	15.1	
Clinical type							0.237
TT	418	17.8	312	18.8	106	15.4	
BT	440	18.7	313	18.8	127	18.4	
BB	164	7.0	113	6.8	51	7.4	
BL	735	31.2	502	30.2	233	33.8	
LL	596	25.3	424	25.5	172	25.0	
Operational type							0.167
MB	1927	81.9	1351	81.2	576	83.6	
PB	426	18.1	313	18.8	113	16.4	
Disability							<0.001
no disability	1248	53.1	821	49.4	427	62.0	
G1D	403	17.1	310	18.6	93	13.5	
G2D	702	29.8	533	32.0	169	24.5	

Note: Clinical and operational types of the leprosy patients. TT: tuberculoid; BT: borderline-tuberculoid; BB: mid-borderline; BL: borderline-lepromatous; LL: lepromatous; MB: Multibacillary; PB: Paucibacillary leprosy.

Disability of the leprosy patients. ND: no disability; G1D: grade 1 of disability; G2D: grade 2 of disability.

### Antigen-specific antibody detection by enzyme-linked immunosorbent assay (ELISA)

NDO-BSA and LID-1 were generated at Infectious Disease Research Institute, Seattle, USA and MMP-II was generated at Department of Mycobacteriology, Leprosy Research Centre, National Institute of Infectious Diseases, Japan. ELISA for the detection of antigen-specific antibodies (Abs) was performed in accordance with published procedures^4,6–8^. The cut-off values were determined by Receiver Operating Characteristic (ROC) curve analysis of three replicate experiments as the value providing best overall performance characteristics for each antigen (sensitivity, specificity and area under the curve). The cut-off values were defined as OD450nm of 0.2364, 0.1654 and 0.1384 for NDO-BSA, MMP-II and LID-1, respectively. Detailed protocols and procedures can be found in Supplementary method. All methods were carried out in accordance with relevant guidelines and regulations. All experimental protocols were approved by the Ethics Committee in institute of dermatology, Chinese academy medical science. Informed consent was obtained from all patients and healthy volunteers before blood was collected.

#### Statistical analysis

Person-years (PY) were calculated through approximate method and the incidence rates of leprosy among HHCs or the general population were expressed in person-years (PY) after the follow-up (0.1 to 4.5 years), taking into account person lost to follow-up, diseased or deceased. Distributed continuous data were expressed as mean ± SD. Student’s t-test was used to compare parametric continuous data between groups and the chi-square test was used for the categorical data. Spearman correlation coefficient was also calculated using SPSS 17.0 software. The p value < 0.05 was considered as statistically significant.

## Results

### Characteristics of leprosy patients in southwest China

**Figure 2:**
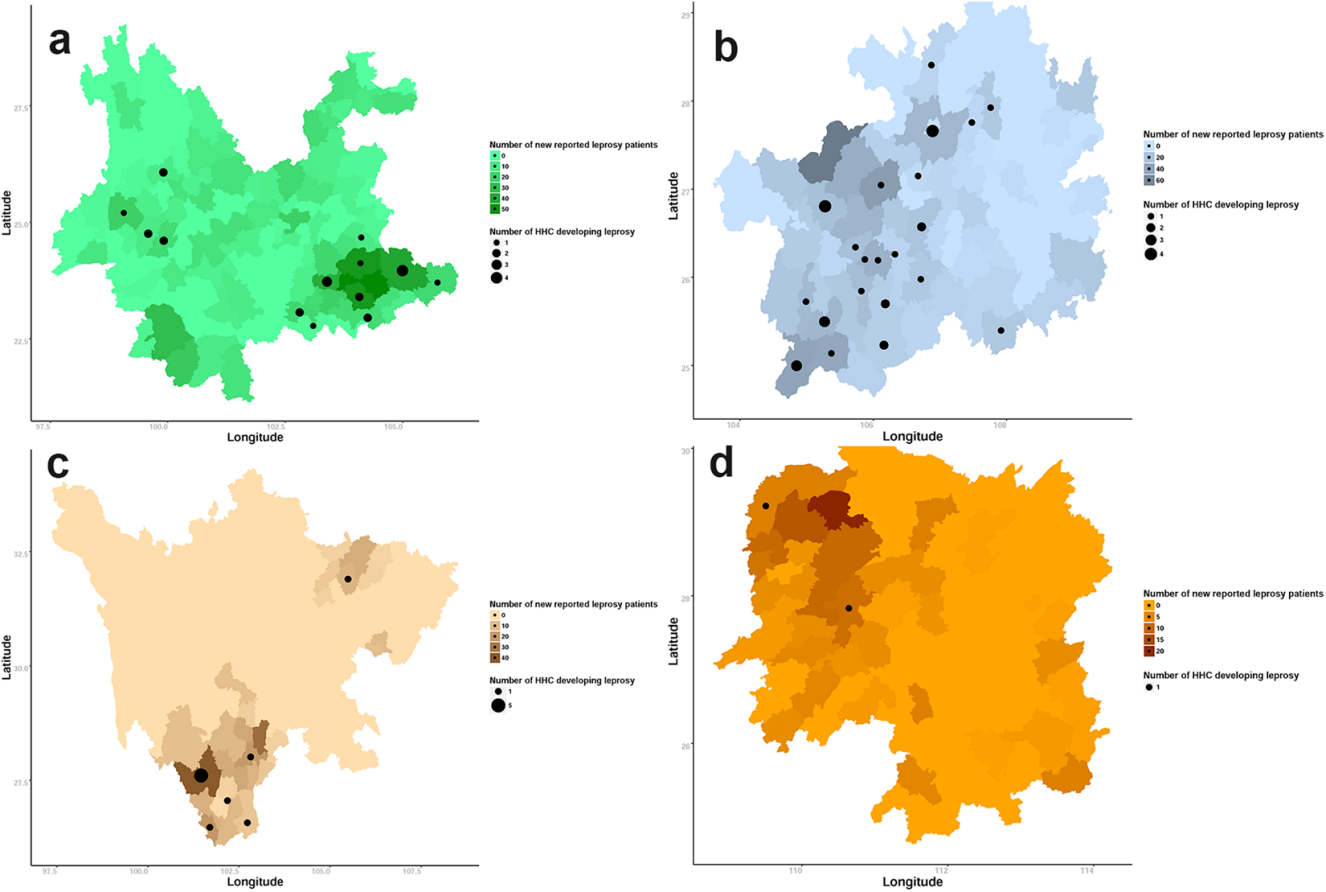
Geographic distribution of new leprosy cases, including HHCs that developed leprosy, in southwest China between January 2010 and June 2014. In (**a**), Yunnan, (**b**) Guizhou, (**c**) Sichuan and (**d**) Hunan are highlighted with different colors to represent the number of new leprosy cases reported among these four provinces. The depths of colour represent the number of new leprosy patients. The location of each patient recorded, and HHC that developed leprosy, in the monitoring period in (**a**) Yunnan, (**b**) Guizhou, (**c**) Sichuan and (**d**) Hunan provinces are depicted by black dots, with the size of each dot representing the number of HHC that developed leprosy. The maps in this figure were generated by means of software R, including maptools, maps and mapdata (Version 3.3.3, https://cran.r-project.org/src/base/R-3/).

The total study population recruited had a mean age ± SD was 45.42 ± 16.34 years, consistent with the mean age ± SD of the patients at diagnosis, which was 42.26 ± 16.28 years. A total of 2353 leprosy patients were enrolled during the study period (January 2010 to June 2014; 956 from Yunnan, 865 from Guizhou, 348 from Sichuan and 184 from Hunan) (Fig. 1). Among these 4 provinces, Yunnan reported with the highest number of leprosy patients in these four years, with the majority of cases located in the Wenshan and Honghe prefectures of south-eastern Yunnan (Fig. 2a). Western Guizhou, Hunan, and Southern Sichuan yielded the greatest number of patients in each of the corresponding provinces (Fig. 2b–d).

Patient characteristics, clinical diagnosis and selected medical disorders were evaluated by doctors and local staff (Table 1). Individuals with indeterminate clinical types or unclear disabilities were excluded from the study. The patient population comprised 1664 males and 689 females, demonstrating the disease skewed toward males. Differences in age at the time of survey and the disease diagnosis were not observed between the genders (p = 0.346 and 0.40, respectively). Although no significant differences were found in age distribution, clinical type and operational classification (p = 0.137, 0.237 and 0.167, respectively), a higher proportion of grade 2 disabilities (G2D) were observed among male patients (p < 0.001 versus female patients). Different constituent ratios of disability were noted among the different clinical types (p < 0.001; Table 2). The highest ratio of G2D was recorded among patients of the T-lep (tuberculoid leprosy, including both TT and BT type).

**Table 2:** Relationship between clinical types and disability.

	T-lep		BB		L-lep		p-value
	N	%	N	%	N	%	
No disability	372	43.4	95	57.9	781	58.7	<0.001
G1D	111	12.9	30	18.3	262	19.7	
G2D	375	43.7	39	23.8	288	21.6	

### Impact of local natural and economic conditions on leprosy incidence

**Figure 3:**
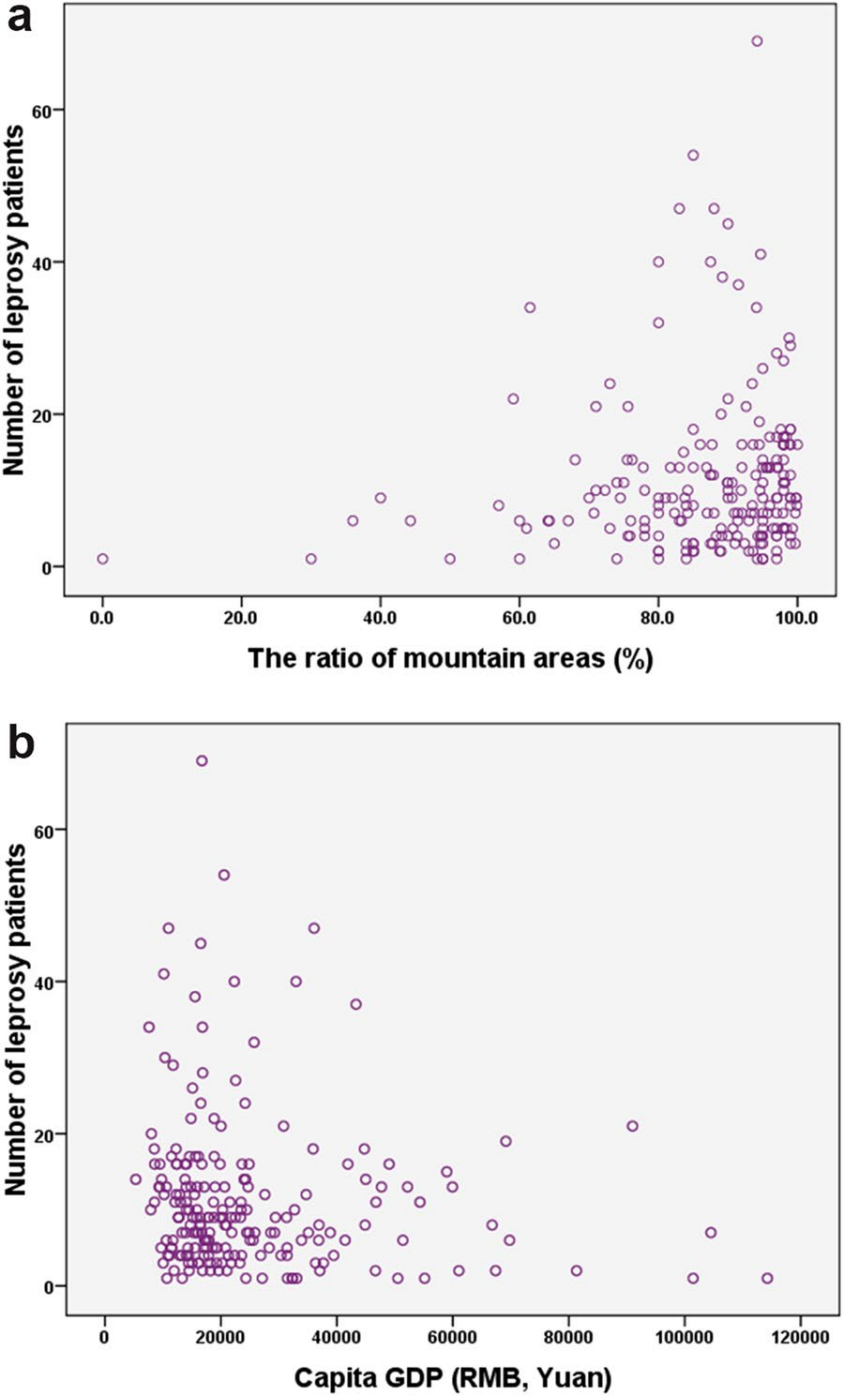
Impact of socioeconomic conditions on leprosy incidence. In (**a**) local natural and (**b**) economic conditions are plotted against the number of patients. Each point represents an individual county.

After acquiring the data of each county from the departments of statistics, we used the ratio of mountain areas and capita GDP as a proxy of the local natural and economic conditions. We then analysed the relationships between these data and the distribution of leprosy cases (Fig. 3). The correlation coefficient between the number of leprosy cases and the ratio of mountain areas was 0.162 (p = 0.02), while the correlation coefficient between the number of leprosy cases and the capita GDP was −0.184 (p = 0.008). The analyses indicate leprosy cases are most common found in poor regions with difficult terrain.

### Incidence of leprosy among HHCs

A total of 6643 HHCs were screened between January 2010 and June 2014, amongst whom 53 developed leprosy during careful follow up of 0.1–4.5 years. We then calculated the incidence of leprosy among HHCs by PY in each of the four provinces. We also identified the number of new leprosy patients amongst the general population in these four provinces during the monitoring period and similarly calculated the incidence of leprosy. The incidence rate of leprosy amongst HHCs was 364/100,000 PY (53/14553 PY), a rate far higher than the incidence of 0.28 per 100,000PY which was measured among the general population in four provinces. These data indicate the risk of HHCs developing leprosy was 1300 times higher than the risk among the general population during this monitoring period.

### Antigen-specific antibodies in leprosy patients

**Figure 4:**
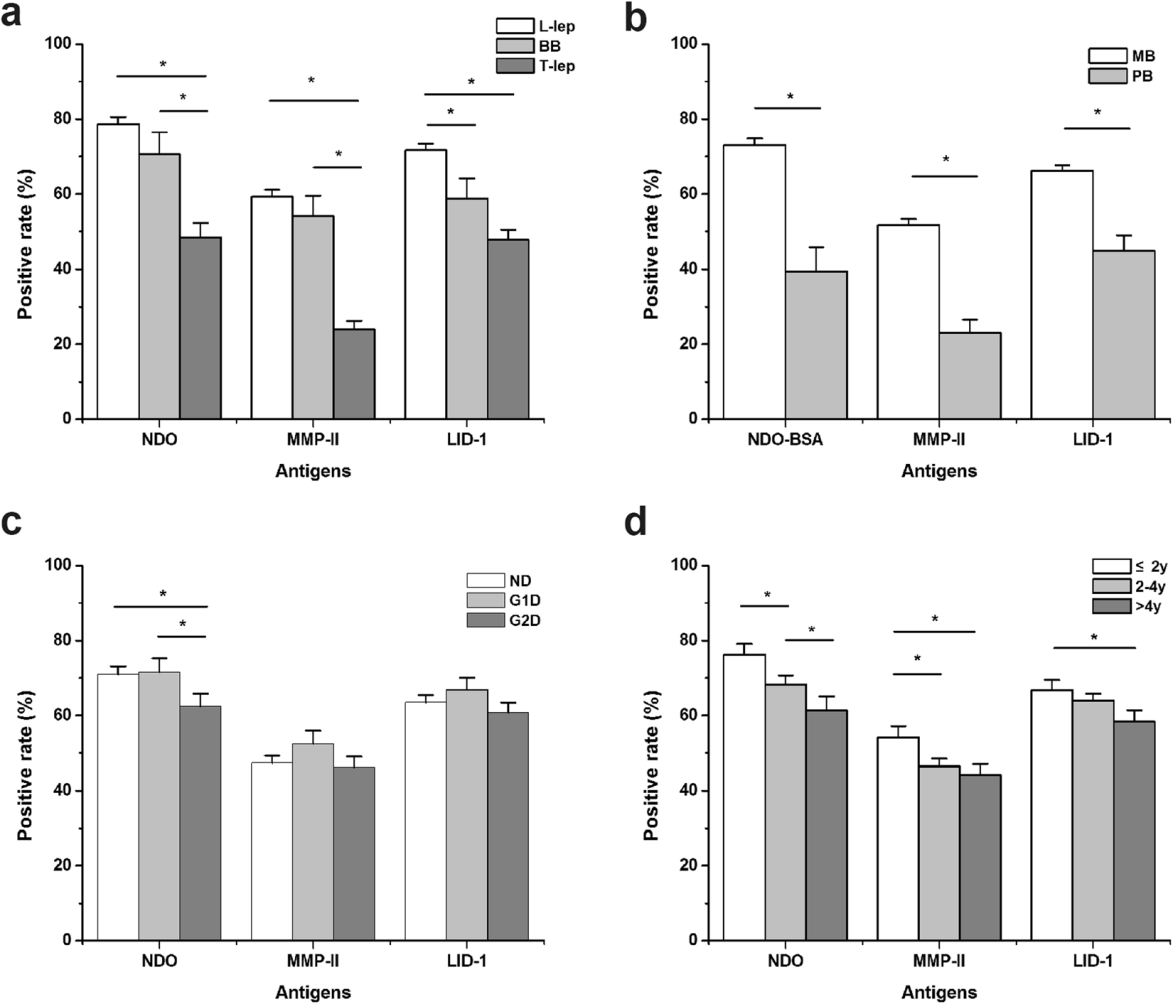
Proportion of patients presenting with antigen-specific antibody responses (NDO-BSA, MMP-II and LID-1). Patients were classified by either (**a**) clinical presentation, (**b**) operational classification, (**c**) disability grade or (**d**) length of time since initial diagnosis. *p-value < 0.05 between the indicated groups.

We also examined serum antibody responses of each patient to generate a simple immune profile and determine how this related to clinical presentation. Sera from 1122 patients were randomly selected for serological evaluation using all three antigens, while sera from the other 1231 patients were evaluated by one or two antigens (results were similar for each individual antigen evaluated between the cohorts). As expected, the proportion of patients with circulating antibodies against the three antigens differed between operational classification and across the clinical types (Fig. 4a,b and Supplementary Table S1). L-lep patients had the highest seropositivity rates at 78.7%, 59.3% and 71.7%) against NDO-BSA, MMP-II and LID-1, respectively, while the seropositivity rates among T-lep patients were lower, especially for MMP-II (23.9%). Significant differences were observed between L-lep and T-lep type for each of the antigens (all p = 0.000). The seropositive rates among BB patients ranged between those observed for L-lep and T-lep. The seropositive rates of antibodies against NDO-BSA and LID-1 were higher than those observed against MMP-II.

With regards to presenting with disabilities, the seropositive rates of anti-MMP-II and anti-LID-1 antibodies were not significantly different from each other in patients with G1D and G2D. The proportion of patients seropositive for antibodies against NDO-BSA was, however, slightly but significantly decreased in G2D leprosy patients relative to those patients without disability or with G1D. No significant differences were observed for these disability grades when assessing antibodies against LID-1 or MMP-II (Fig. 4c and Supplementary Table S1).

Consistent with a decline in antibody levels during and after completion of MDT, we found that the highest seropositivity rates were measured in patients within the initial 2 years of their diagnosis and this gradually fell among patients further removed from the time of diagnosis/completion of MDT (Fig. 4d and Supplementary Table S1).

Using the accumulation of seropositive responses against any of the three antigens evaluated, a total of 1122 patients (MB:975, PB:147) had been detected with only 11.6% MB patients displaying negative responses to all three antigens (Table 3). Similar analysis indicated that 40.1% of PB patients could not be detected using all combinations of antigens.

**Table 3:** Combination of different antigens to detect antileprosy antibodies in leprosy patients.

Antigens		MB	PB
		N(%)	N(%)
All positive		354(36.3%)	19(12.9%)
At least one positive	NDO-BSA-MMP-II	778(79.8%)	70(47.6%)
	NDO-BSA-LID-1	841(86.3%)	83(56.5%)
	MMP-II-LID-1	751(77.0%)	74(50.3%)
All negative		113(11.6%)	59(40.1%)
Total		975	147

## Discussion

This work described the epidemiological features of leprosy in southwest China and determined the presence of antigen-specific serum antibodies amongst leprosy patients with different clinical types and disease severity. Our large sample sizes enabled the accurate estimation of epidemiological characteristics. Data analyses revealed some pockets of patients across the 4 provinces, namely Wenshan and Honghe in Yunnan; Bijie and Qianxinan in Guizhou; Liangshan in Sichuan, and Xiangxi and Huaihua in Hunan, that were inhabited by many minority inhabitants. These areas currently have poor general living environments, poor transportation systems and weak economies. Thus, in addition to thorough monitoring, more government financing to develop the conditions in these particular pocket areas would be helpful with regard to leprosy control. We believe the leprosy situation will continuously improve as the local economy develops^9,10^.

Index cases are believed to be the main source of *M*. *leprae* infection and prolonged contact with a patient is a known risk factor for its transmission^11^. Through screening of 42113 persons in India, Kumar *et al*. previously reported that the incidence rate of leprosy in familial contacts was significantly higher than in nonfamilial contacts (676 versus 46/100,000 PY)^12^. A survey in Qianxinan prefecture, Guizhou province, found that 190 of 542 new patients had an association with families with a known history of leprosy, giving a constituent ratio of 35.1%^10^. Thorough understanding of the incidence rate of leprosy among HHC in China is, however, still lacking. Our data indicate in southwest China that the incidence rate of leprosy in HHCs was 364/100,000 PY, a rate far higher than that observed in the general population (0.28/100,000 PY). It is noteworthy that a study in the general population of a highly endemic area in Bangladesh reported the incidence rate of leprosy as 37/100,000 PY over six years^13^, more than 100 times the incidence rate among the general population in southwest China. The discrepancy of incidence rates is likely due to the local endemicity: the India and Bangladesh studies were conducted in high endemic regions, while our overall study areas are considered as relatively low endemic areas. In view of these data, we support a focus on two key overlapping groups for leprosy control: areas with pockets of index cases and HHC.

Official reports indicate that the ratio of male to female patients is often skewed: 2.3:1 in China^14,15^, 2.27:1 in Philippines^16^ and 0.53:0.47 in Brazil^17^. In the present study of southwest China we observed a bias in male and female ratio to 2.42:1, well in agreement with national records. This appears to indicate that males in these provinces are more prone to the development of leprosy than females. The reason behind this is not yet known.

Among 5 types of leprosy patients as per the Ridley–Jopling’s classification^18^, we noticed, L-lep patients (including BL and LL) cover the majority (56.5%) of the clinical types. Refer to WHO classification, the proportion of MB and PB leprosy patients are 81.9% and 18.1% respectively, which was constant occurrence since 2001 in China^15^. T-lep patients recorded higher rate of grade 2 disability than L-lep and BB type (Table 2), might be because that T-lep patients showed specific cell-mediated immunity against *M*. *leprae*. Although existence of very low number of bacilli in T-lep stage, it can be able to induce granulomatous inflammatory response to *M*. *leprae* and lead to sensory and motor neuropathies^1^.

We have previously used some protein antigens in serological diagnosis of leprosy^4^. In this study we used three antigens namely, NDO-BSA, MMP-II (major membrane protein-II) and LID-1 (Leprosy IDRI Diagnostic-1), to detect circulating antigen-specific antibodies in leprosy patients. Among the three antigens, NDO-BSA yielded the highest seropositive rate across all leprosy presentations, although LID-1 yielded a higher seropositive rate among PB patients. The previously reported seropositive rates for MMP-II were reported as 82.4% in MB and 39.0% in PB leprosy patients by Yumi *et al*.^19^, and 98% and 48% as described by Hatta *et al*.^20^. The rates are lower in the current study, and our previous evaluations used similar samples from southwest China, however, and showed positive rates of 88.1% and 61.1%^4^. This discrepancy may be due to differences among regions or simply a function of the greater number and diversity of samples (some collected from patients during and after MDT) analysed in this study. Indeed, we observed that seropositive rates for antibodies against NDO-BSA gradually decreased since the treatment but a significant drop was only prominent in 4 years for MMP-II, and no significant decline in seropositivity was observed for antibodies against LID-1 in 4 years.

Duthie^17^ reported a new leprosy serological test involving NDO directly conjugated to the LID-1 protein (NDO-LID) detected larger proportions of MB and PB leprosy than the alternative Standard Diagnostics leprosy test using only NDO-BSA (87.0% versus 81.7% and 32.3% versus 6.5%, respectively). Our data indicate that a combination of responses against these antigens and MMP-II could detect the greatest number of leprosy patients, indicating further complementation. Our former work reported that the combination of NDO-BSA and MMP-II could detect all MB patients and 72.2% of PB patients^17^, in the current study these numbers were 79.8% and 47.6%, respectively. Again, with almost 30 times the number of samples in this current study, the difference likely arises from the total number evaluated. The combination of NDO-BSA and LID-1 was more effective to detect leprosy patients in the current study, which detecting 86.3% MB and 56.5% PB. Taken together, our data provides further indication that combinations of different antigens could assist in the diagnosis and monitoring of leprosy.

In conclusion, based upon our observations and data generated, we recommend the application of serologic assays detecting antibodies against multiple antigens for effective coverage and monitoring in leprosy control. Particular emphasis should be placed on attending to HHCs within the leprosy pockets in southwest China as a means to provide early diagnosis and treatment, as a strategy to disrupt the transmission of *M*. *leprae* and provide more precise control of the leprosy situation.

## Electronic supplementary material

Supplementary method and table S1
