# Supplementary material for: Monitoring and detection of leprosy patients in Southwest China: A retrospective study, 2010–2014
Source: Sci Rep. 2018 Jul 30;8:11407. doi: 10.1038/s41598-018-29753-4 (PMC6065315; doi:10.1038/s41598-018-29753-4)
Supplement: Supplementary file 1 — Supplementary method and table S1 [file 41598_2018_29753_MOESM1_ESM.docx]

**Monitoring and detection of leprosy patients in Southwest China: A retrospective study, 2010-2014**

Wang Le^a,b^, Jiang Haiqin^a,c^, Hao Danfeng^a^, Shi Ying^a^, Zhang Wenyue^a^, Yang Jun^d^, XiongLi^d^, ShuiTiejun^d^, Shen Limei^e^, Liu Jie^e^, Wang De^e^, Ning Yong^f^, Liu Yangying^f^, Wang Hao^f^, KuangYanfei^g^, Li Bin^g^, Yumi Maeda^h^, Malcolm Duthie^i^, Yu Meiwen^a,b^, Wang Hongsheng^a,b,c^, Yan Liangbin^a,b^, Zhang Guocheng^a,b^, Wang Baoxi^a,b^& Gu Heng^a,b^.

**Supplementary material**

- Supplementary method
- Supplementary table

**Supplementary method. Determination of cut-off values for antigen-specific antibody detection by ELISA.**

We got the three cut-off values for NDO-BSA, MMP-II and LID-1 according the following procedure. Firstly, the OD values of leprosy patients and healthy control were inputted into SPSS software, and then the data were analyzed by the function of ROC curve. Finally, we got the corresponding ROC curve. We could calculate the Youden's index and confirm the cut-off value. We got the largest Youden’s index when the OD value for NDO-BSA was 0.2364, meanwhile the sensitivity was 91.5%, specificity was 91.5%, and area under the curve was 0.951. As to NDO-BSA, we also got the cut-off value for MMP-II when the OD value was 0.1654, meanwhile the sensitivity was 83%, specificity was 100%, and area under the curve was 0.953. We also got the cut-off value for LID-1 when the OD value was 0.1384, meanwhile the sensitivity was 87.2%, specificity was 91.5%, and area under the curve was 0.93.

**Supplementary table S1. Positive rates of antigens under different conditions of clinical or operational types, disability and time**

|  | **Clinical type** | **NDO-BSA** | **MMP-II** | **LID-1** |  | ***p*-value** | | | |
| --- | --- | --- | --- | --- | --- | --- | --- | --- | --- |
|  |  | **N(%)** | **N(%)** | **N(%)** |  | **Total** | **NDO-BSA versus MMP**-II | **NDO-BSA versus LID-1** | **MMP-II versus LID-1** |
| **Clinical type** | L-lep | 543(78.7%) | 409(59.3%) | 495(71.7%) |  | 0.000 | 0.000 | 0.003 | 0.000 |
|  | BB | 60(70.6%) | 46(54.1%) | 50(58.8%) |  | 0.076 | 0.027 | 0.109 | 0.536 |
|  | T-lep | 168(48.4%) | 83(23.9%) | 166(47.8%) |  | 0.000 | 0.000 | 0.879 | 0.000 |
| **Operational type** | MB | 713(73.1%) | 504(51.7%) | 645(66.2%) |  | 0.000 | 0.000 | 0.001 | 0.000 |
|  | PB | 58(39.5%) | 34(23.1%) | 66(44.9%) |  | 0.000 | 0.003 | 0.345 | 0.000 |
| **Disability** | No disability | 433(70.9%) | 289(47.3%) | 388(63.5%) |  | 0.000 | 0.000 | 0.006 | 0.000 |
|  | G1D | 149(71.6%) | 109(52.4%) | 139(66.8%) |  | 0.000 | 0.000 | 0.288 | 0.003 |
|  | G2D | 189(62.4%) | 140(46.2%) | 184(60.7%) |  | 0.000 | 0.000 | 0.676 | 0.000 |
| **Time**  **(Year)** | ≦2 | 225(76.3%) | 160(54.2%) | 197(66.8%) |  | 0.000 | 0.000 | 0.011 | 0.002 |
|  | 2-4 | 375(68.3%) | 255(46.4%) | 351(63.9%) |  | 0.000 | 0.000 | 0.126 | 0.000 |
|  | >4 | 171(61.3%) | 123(44.1%) | 163(58.4%) |  | 0.000 | 0.000 | 0.490 | 0.001 |
